# Incorporating meteorological factors into a SARIMA model for predicting pediatric influenza epidemics

**DOI:** 10.3389/fpubh.2026.1776470

**Published:** 2026-04-08

**Authors:** Shiyin Mu, Ruiwen Xia, Jia Zhai, Yongsheng Guo, Jiao Li, Mei Yu, Run Guo, Xingda Wen, Yingxue Zou, Wei Liu

**Affiliations:** 1 Department of Respiratory of Ma-Chang, Children's Hospital, Tianjin University/Tianjin Children's Hospital, Tianjin, China; 2 Clinical School of Paediatrics, Tianjin Medical University, Tianjin, China; 3 Children's Hospital, Tianjin University/Tianjin Children's Hospital, Tianjin, China

**Keywords:** children, GAM model, influenza, meteorological parameters, SARIMA model

## Abstract

**Objective:**

Influenza, a contagious respiratory illness, imposes a substantial global disease burden, underscoring the critical need for timely and accurate surveillance systems. Seasonal Autoregressive Integrated Moving Average (SARIMA) models excel in capturing seasonal patterns and trends of infectious diseases. The distinct seasonality of influenza suggests that meteorological factors significantly influence its transmission, though their specific mechanisms remain incompletely characterized. We integrated meteorological variables into a SARIMA model to enhance the prediction of pediatric influenza epidemics, thereby providing a scientific foundation for precise prevention and control strategies.

**Methods:**

We analyzed 67,770 influenza-like illness (ILI) cases from Tianjin Children’s Hospital (June 2018–June 2023) alongside meteorological data (temperature, humidity, precipitation, atmospheric pressure, wind speed, sunshine duration). We characterized influenza epidemiology, employed Spearman correlation and generalized additive models (GAM) to quantify associations between meteorological factors and ILI, and developed SARIMA models to forecast epidemic trends.

**Results:**

Pediatric influenza in Tianjin demonstrated significant seasonal and spatial heterogeneity, with children under 6 years comprising >70% of cases. Spearman analysis revealed negative correlations between ILI cases and relative humidity (*r* = −0.31), temperature (*r* = −0.30), and precipitation (*r* = −0.36) (all *p* < 0.05), while atmospheric pressure exhibited a positive correlation (*r* = 0.34, *p* < 0.05). GAM identified relative humidity as the most influential meteorological factor (*F* = 2.40, *p* = 0.00478). The SARIMA(1,0,0)(0,0,0)_12_ model demonstrated robust performance (*R*^2^ = 0.632, RMSE = 27.33).

**Conclusion:**

Pediatric influenza in Tianjin predominantly affects children under 6 years, peaks in winter, and clusters in urban centers. Low-temperature and low-humidity conditions−particularly low relative humidity−exacerbate transmission. SARIMA models incorporating meteorological parameters effectively predict influenza incidence, supporting data-driven public health interventions.

## 1 Introduction

Influenza is an acute respiratory infection caused by influenza viruses and is characterized by rapid transmission (1). Influenza, as a global public health problem, causes about 3–5 million severe cases and 290,000–650,000 influenza-associated respiratory deaths each year (2), a number that increases dramatically when a pandemic occurs. Children, due to their immature immune system and aggregated activity characteristics, can be infected at a rate of 20–30% and have a 4–7 times higher risk of hospitalization than adults (3). Influenza is usually most prevalent in winter and spring, but recent reports have shown a significant surge in influenza cases during the summer months (4), an emerging trend that poses new challenges for health authorities and influenza surveillance.

Influenza outbreaks exhibit distinct seasonal patterns, and an increasing number of studies suggest that meteorological factors also play a key role in the transmission of influenza (5). These factors can influence influenza occurrence by affecting pathogen viability and spread, human behavior, and host immunity, with mechanisms showing significant regional variation. Studies in temperate zones suggest low temperatures promote transmission by extending viral survival (6), whereas in tropical regions, high temperatures and humid conditions during rainy seasons may enhance viral activity (7). Additionally, Shi et al. (8) found that the duration of influenza epidemics is longer in mid-latitude regions, which is related to the influence of climate factors such as daylight hours on influenza virus transmission. Qi et al. (9) discovered that in Chongqing, when wind speeds are below 1.55 m/s, the risk of influenza activity significantly increases, and this risk can persist for 4 weeks, indicating that low wind speeds are an important meteorological factor in influenza transmission.

To effectively mitigate large-scale outbreaks and reduce influenza burden, establishing timely and accurate surveillance systems is crucial. Significant progress has been made in recent years utilizing various mathematical models for infectious disease early warning and prediction, including logistic regression models (10), Autoregressive Integrated Moving Average (ARIMA) models (11), and Long Short-Term Memory (LSTM) neural networks (12). Notably, the SARIMA model excels in accurately identifying seasonal patterns and trends in infectious diseases (13, 14). However, existing research often overlooks the broader impact of meteorological factors, typically focusing on individual parameters and lacking a systematic analysis of the multidimensional influences of weather on childhood influenza. Integrating meteorological factors into SARIMA models holds promise for more effective influenza incidence prediction.

Children bear 40% of Tianjin’s influenza burden, with infants under 3 years experiencing severe complications in 12% of cases. To investigate the meteorological drivers of this burden, we analyzed correlations between weather patterns and influenza transmission dynamics, developing integrated SARIMA models that incorporate meteorological variables to forecast epidemic trends. This provides precision tools for targeted control strategies and optimizes resource allocation—particularly vaccination programs—to alleviate pediatric healthcare burdens.

## 2 Materials and methods

### 2.1 Study area

Tianjin, one of China’s four direct-controlled municipalities, is situated between 38°34′–40°15′N and 116°43′–118°04′E within the UTC + 8 time zone. Administratively, the city comprises 16 districts categorized as: six urban districts (Heping, Hedong, Hexi, Nankai, Hebei, Hongqiao); four peripheral districts (Dongli, Xiqing, Jinnan, Beichen); five suburban districts (Wuqing, Baodi, Jinghai, Ninghe, Jizhou); and Binhai New Area. Geographically positioned on the northeastern North China Plain at the confluence of the Haihe River’s five tributaries, Tianjin features a warm-temperate semi-humid monsoon climate characterized by four distinct seasons: hot, humid summers with concentrated rainfall; and cold, dry winters with frequent climatic variability.

### 2.2 Data collection

ILI case data were extracted from the outpatient and emergency department records of Tianjin Children’s Hospital covering the period June 2018 to June 2023. ILI cases were defined based on standard clinical criteria (15): a body temperature of ≥38 °C accompanied by either a cough or sore throat, with no alternative diagnosis. Sore throat was specifically defined by clinical observation of pharyngeal inflammation or erythema by a physician. While this definition may include other respiratory viruses, it is the established surveillance case definition for influenza-like illness and has been validated to correlate with influenza virus activity during epidemic periods. A total of 67,770 pediatric cases were included in the study. Collected data encompassed demographic information (sex, age, residence) and temporal information (date of visit).

The meteorological data used in this study were provided by Tianjin Meteorological Service. The climate factors included monthly averages (or totals) for key factors across all 16 districts and the city-wide level:atmospheric pressure (mmHg), wind speed (m/s), air temperature (°C), relative humidity (%), sunshine duration (h), cumulative precipitation (mm).

### 2.3 Methods

#### 2.3.1 Spearman correlation analysis

The number of ILI cases at Tianjin Children’s Hospital from June 2018 to June 2023 was provided by the hospital’s case record system. Spearman correlation analysis was then conducted to examine the relationship between the monthly number of ILI cases and various meteorological factors during the same period, including average atmospheric pressure, wind speed, temperature, humidity, sunshine duration, and total monthly precipitation. This analysis aimed to explore the potential associations between meteorological variables and the incidence of ILI in Tianjin. To account for potential delays between meteorological exposure and clinical presentation, we performed Spearman correlation analysis at lags of 0 to 4 months (Lag0–Lag4). The lag with the strongest correlation was subsequently used in the GAM and SARIMA modeling.

#### 2.3.2 Generalized additive model

The Generalized Additive Model (GAM) is a flexible regression method that models nonlinear relationships between input variables. It enables effective analysis of the complex associations between meteorological factors and influenza activity. Unlike traditional linear regression models, GAM allows each predictor variable to affect the dependent variable in a nonlinear way. This feature helps to capture the potential impact of meteorological variations on the ILI cases. Specifically, the GAM models the relationship between meteorological variables (such as temperature and humidity) and ILI case data. It identifies patterns of influenza activity under different weather conditions, providing more accurate references for influenza forecasting. The modeling formula of the GAM is as follows (1):


$$y=\beta_{0}+\sum_{i=1}^{p}f_{i}(X_{i})+\varepsilon$$ (1)

Here, y represents the number of outpatient visits; $X_{i}$ denotes different predictor variables (meteorological factors); $f_{i}(X_{i})$ is the smooth function of the predictor variables; $\beta_{0}$ is the intercept term; and ε represents the error term.

Based on ILI case data and meteorological data from June 2018 to June 2023, our study developed both univariate and multivariate GAM models. These models not only clearly demonstrated the effects of individual meteorological factors on influenza activity, but also allowed for an in-depth exploration of the combined effects among different meteorological variables by incorporating interaction terms.

#### 2.3.3 SARIMA model

To analyze the temporal dependence and seasonal characteristics of influenza incidence trends, our study employed the Seasonal Autoregressive Integrated Moving Average (SARIMA) model for modeling and forecasting. The SARIMA model is an extension of the ARIMA model and is particularly suited for time series data with distinct seasonal patterns (16). It breaks down the time series into two components: a non-seasonal component (including autoregressive (AR), differencing (I), and moving average (MA) terms) and a seasonal component (including seasonal autoregressive (SAR), seasonal differencing (SI), and seasonal moving average (SMA) terms).

By applying both seasonal and non-seasonal differencing to the ILI incidence data, the SARIMA model can effectively capture temporal variations and identify seasonal fluctuation patterns in influenza incidence. The standard form of the SARIMA model is as follows (2):


$$\Phi_{P}(B^{s})\;\phi_{p}(B)\;\nabla^{d}\nabla_{s}^{D}y_{t}=\Theta_{Q}(B^{s})\;\theta_{q}(B)\;\varepsilon_{t}$$ (2)

Here, $y_{t}$ represents the original value of the time series; $\varepsilon_{t}$ is the white noise error term; B denotes the backshift operator; $\nabla_{s}^{D}$ and $\;\nabla^{d}$ are the seasonal and non-seasonal differencing operators, respectively; $\;\Phi_{P}(B^{s})\;$ and $\phi_{p}(B)\;$ represent the seasonal and non-seasonal autoregressive polynomials, respectively; $\Theta_{Q}(B^{s})$ and $\theta_{q}(B)\;$ denote the seasonal and non-seasonal moving average polynomials, respectively.

Our study established and trained the SARIMA model based on actual observed data from June 2018 to June 2023. The modeling process included differencing for stationarity, model identification, parameter estimation and selection, as well as residual analysis and diagnostic testing. Then, the optimal model was used to forecast the influenza epidemic trends from July 2023 to January 2025.

### 2.4 Ethical considerations

Ethical approval was given by the Medical Ethics Committee of Tianjin Children’s Hospital with the reference number: 2024-LXKY-023. The requirement for written informed consent was waived due to the retrospective nature of the initial analysis, with all patient data anonymized prior to evaluation.

## 3 Results

### 3.1 Analysis of influenza epidemiological characteristics

#### 3.1.1 Spatial distribution

**Figure 1:**
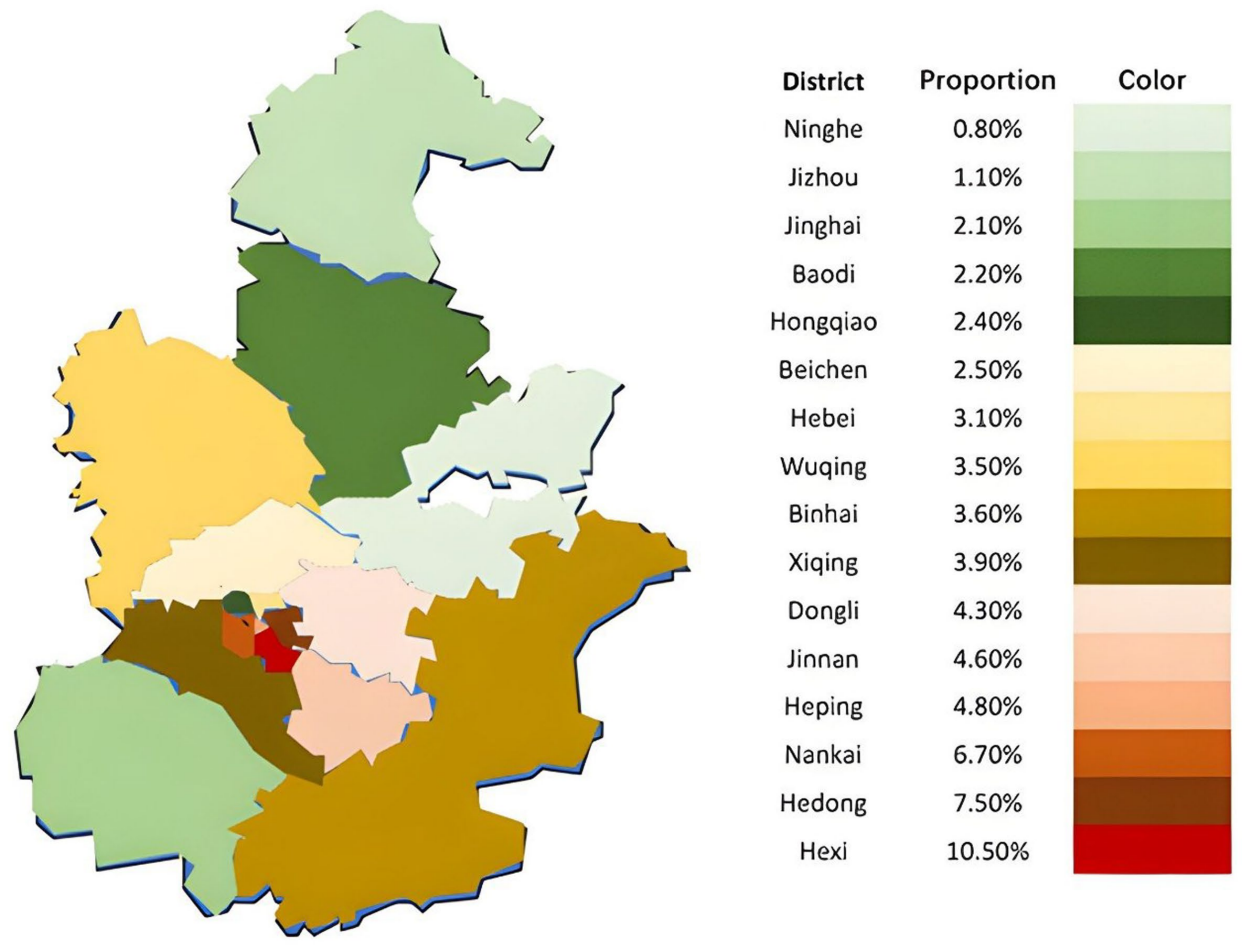
Spatial distribution of the proportion of influenza-like illness cases received by Tianjin Children’s Hospital from 2018 to 2023.

A total of 67,770 ILI cases were recorded at the outpatient and emergency departments of Tianjin Children’s Hospital between June 2018 and June 2023. Figure 1 illustrates the proportional distribution of ILI cases originating from each district within Tianjin. The six central urban districts (Hexi, Hedong, Nankai, Heping, Hebei, Hongqiao)—characterized by higher population density and socioeconomic activity—collectively contributed the largest share of ILI cases. Among these, Hexi district exhibited the highest individual proportion (10.5%), followed by Hedong (7.5%), Nankai (6.7%), and Heping (4.8%). In contrast, the suburban districts (Wuqing, Baodi, Jinghai, Ninghe, Jizhou) demonstrated significantly lower proportions of ILI cases compared to the central urban core (Mann–Whitney U test, *p* = 0.0023). Ninghe district had the lowest proportion (0.8%). This pattern reveals a clear spatial gradient, with ILI incidence progressively decreasing from the densely populated urban center towards the suburban periphery (Figure 1).

**Figure 2:**
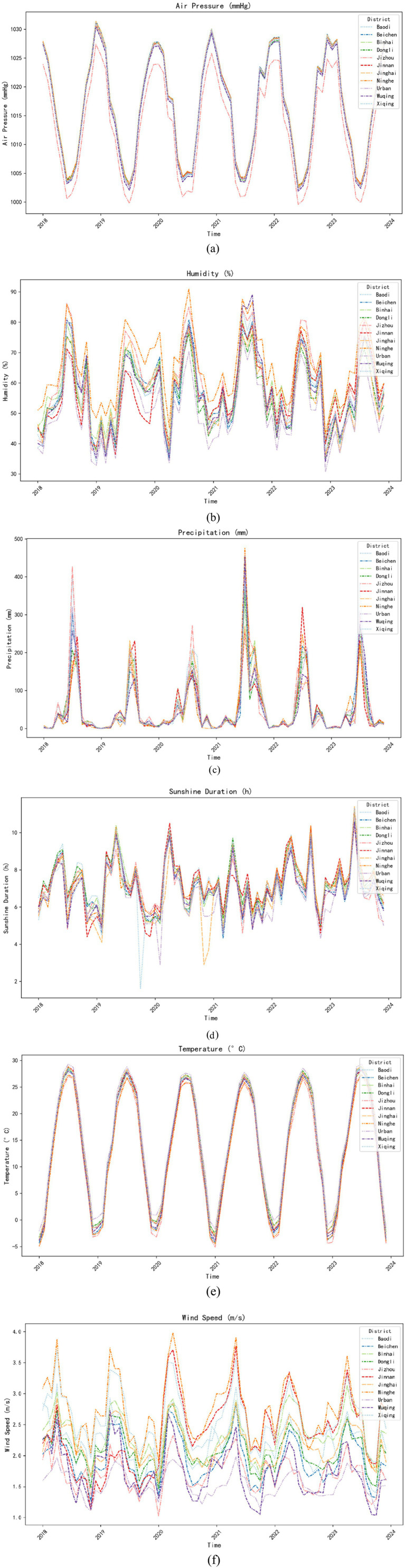
Monthly variation of meteorological factors across districts: urban areas represent the districts of Heping, Hedong, Hexi, Nankai, Hebei, and Hongqiao; **(a)** Air pressure; **(b)** Humidity; **(c)** Precipitation; **(d)** Sunshine duration; **(e)** Temperature; **(f)** Wind speed.

Given the significant spatial heterogeneity in ILI case distribution across Tianjin’s districts, we further examined variations in meteorological factors among these areas. From 2018 to 2023, key meteorological factors in Tianjin exhibited pronounced periodic fluctuations. As shown in Figure 2, monthly variations in these factors demonstrated similar cyclical patterns across all administrative districts. However, the six central urban districts consistently recorded significantly lower values compared to suburban districts for two specific factors: monthly average wind speed and relative humidity.

#### 3.1.2 Temporal distribution

**Figure 3:**
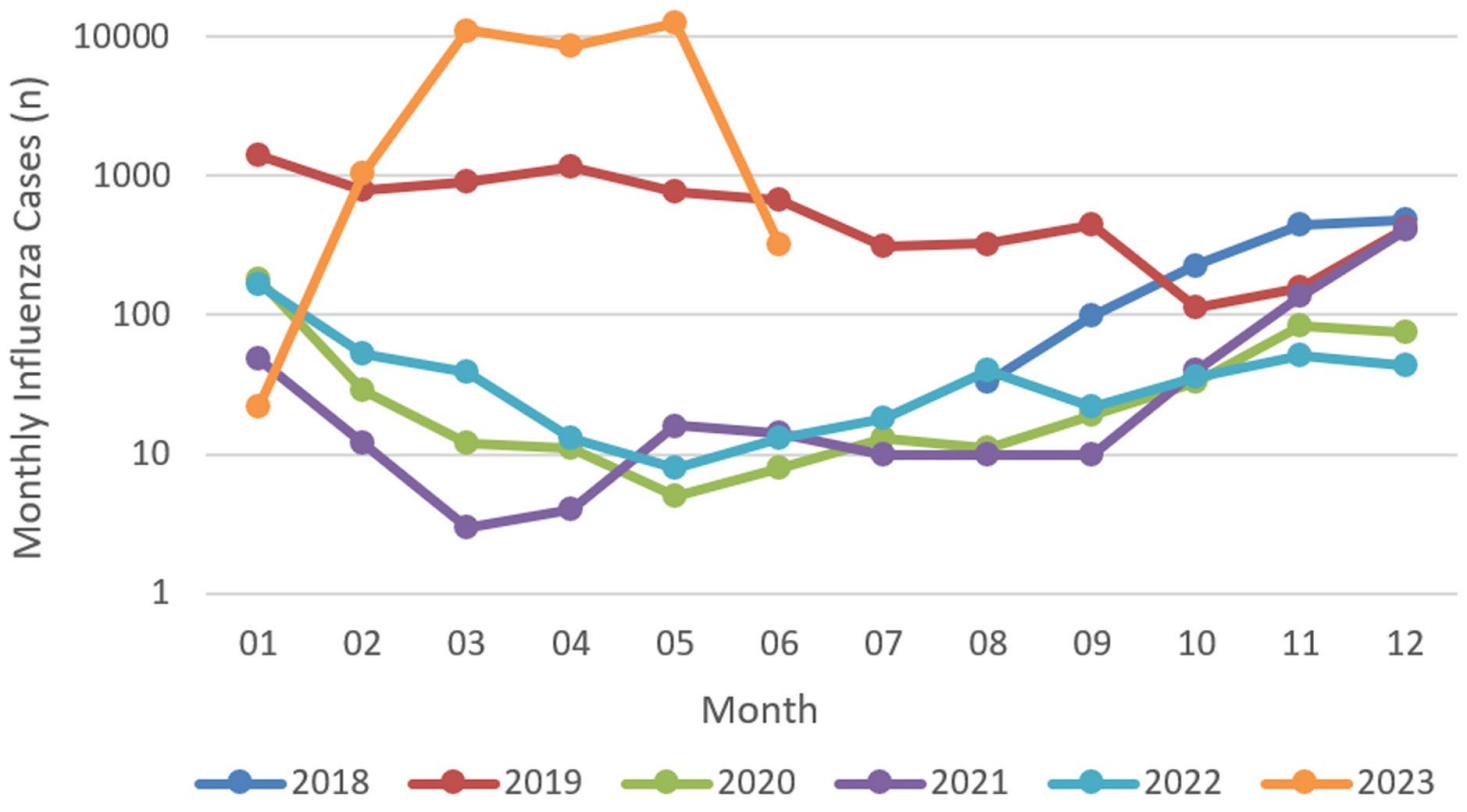
Monthly variation of influenza-like illness cases received by Tianjin Children’s Hospital from 2018 to 2022.

Figure 3 depicts the monthly distribution of pediatric ILI cases at Tianjin Children’s Hospital from 2018 to 2023. The data reveal a consistent seasonal pattern: From 2018 to 2022 (excluding 2019), ILI incidence was relatively low between May and September. Case counts began to rise gradually in October, peaked between December and January of the following year, and subsequently declined. This pattern confirms winter as the primary influenza epidemic season in Tianjin. While 2019 exhibited two distinct peaks in January and April. 2023 saw a significant spring surge, followed by a marked decline in June.

#### 3.1.3 Age distribution

**Figure 4:**
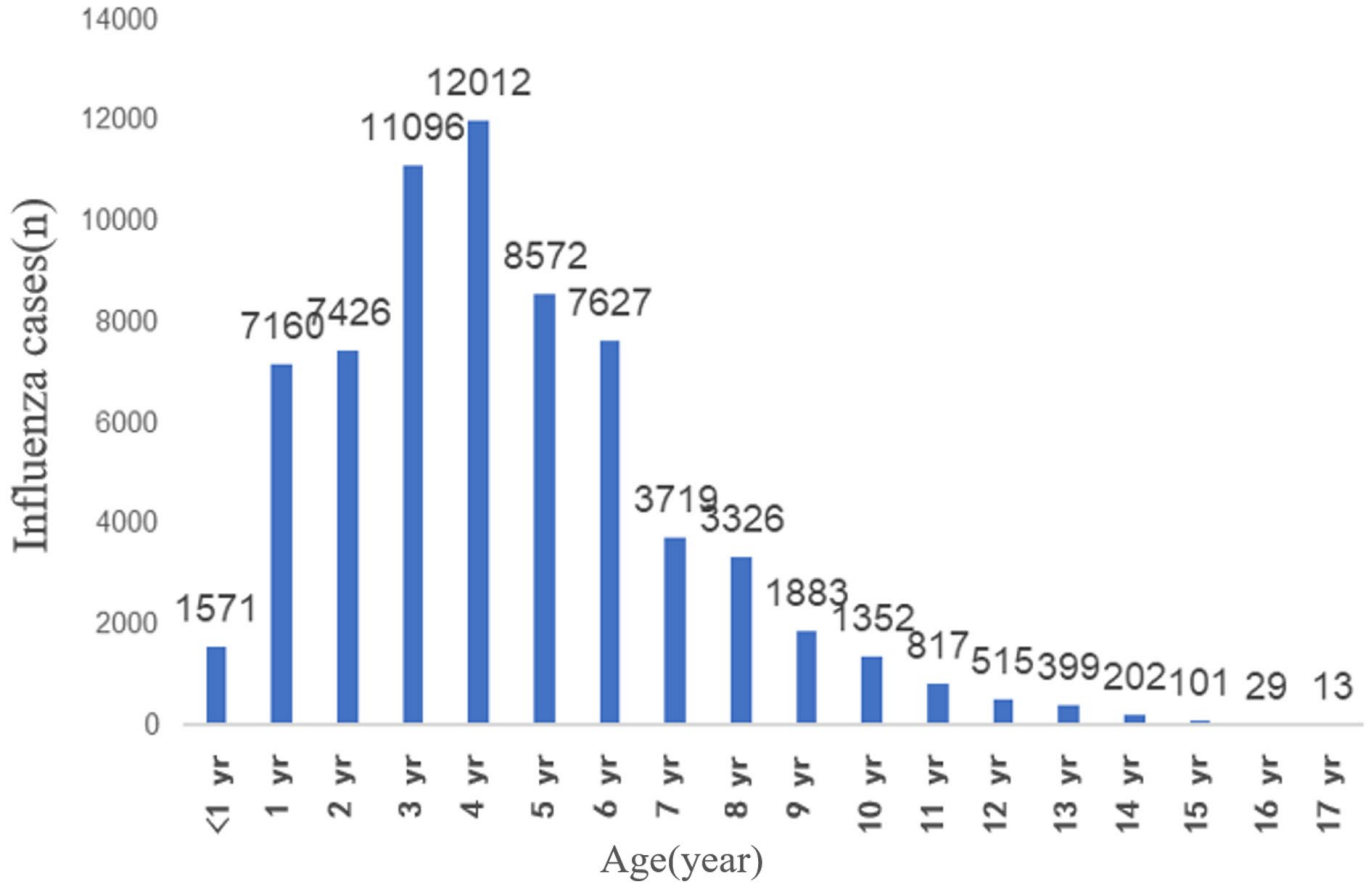
Age distribution of children with influenza-like illness cases received by Tianjin Children’s Hospital from 2018 to 2023.

Figure 4 illustrates the age distribution of the 67,770 ILI cases among pediatric patients treated at Tianjin Children’s Hospital between 2018 and 2023. The patients’ ages ranged from 28 days old to 17 years old, with a median age of 4 years. The cases were categorized by age group as follows:infants and toddlers (<3 years) 16,157 cases (23.84%), preschool children (3–5 years) 31,630 cases (46.75%), school-aged children (6–12 years) 19,239 cases (28.39%), adolescents (13–17 years) 744 cases (1.10%). The data reveals that children under 6 years old accounted for over 70% of the total cases, combining the infants and toddlers and preschool children groups.

### 3.2 Analysis of the relationship between influenza epidemic and meteorological factors

#### 3.2.1 Spearman correlation analysis

**Figure 5:**
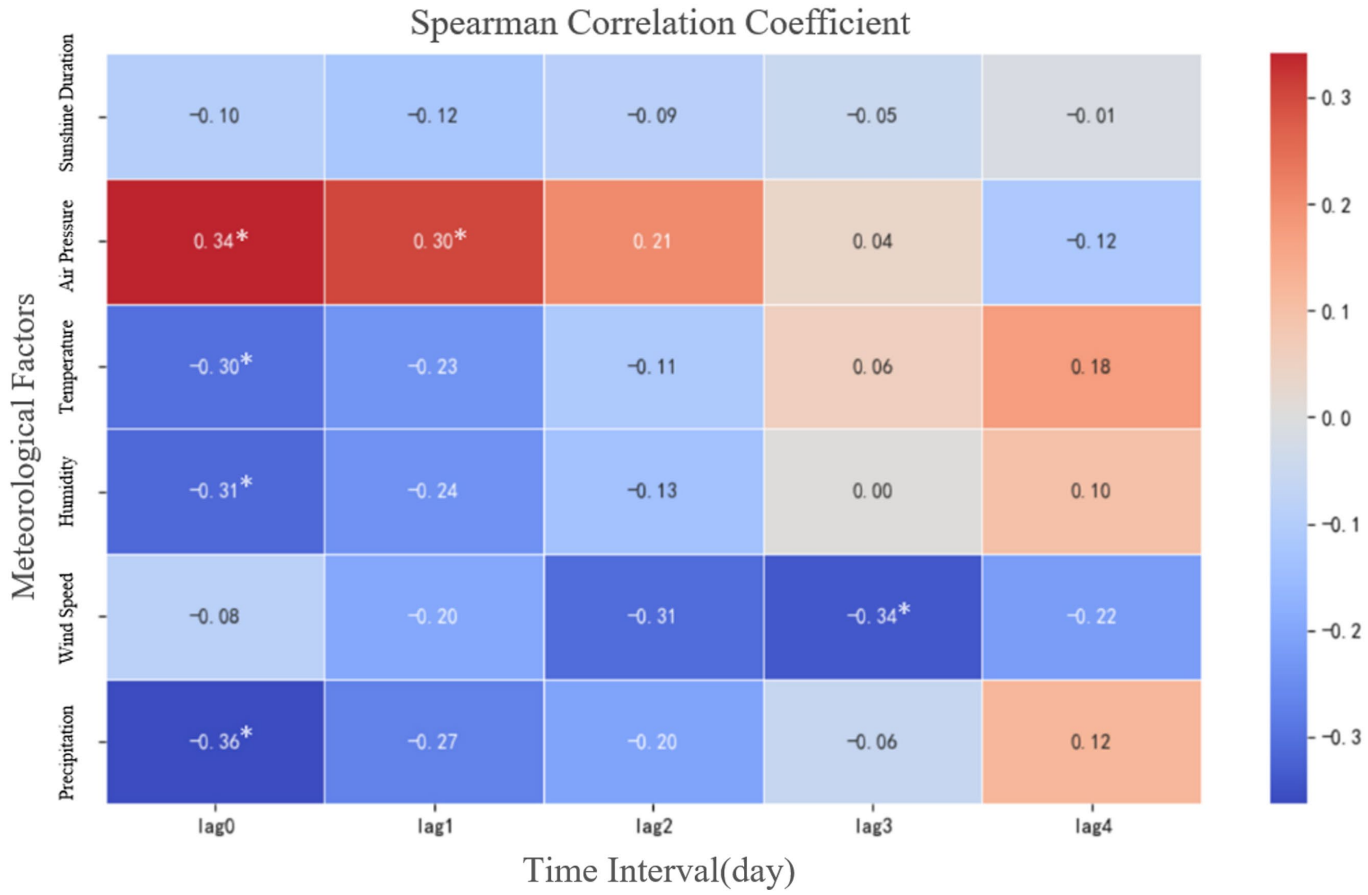
Spearman correlation coefficient plot between meteorological factors and the number of influenza-like illness cases. Lag0–4 represent the time lags (in months) between meteorological factors and influenza-like cases, where Lag0 indicates the current month’s data, and Lag1, Lag2, Lag3, and Lag4 represent data from 1, 2, 3, and 4 months prior, respectively. ‘*’ indicates statistically significant correlations at the 0.05 significance level (two-tailed test).

To investigate the relationships between various meteorological factors and the number of confirmed ILI cases at different lag times, this study employed Spearman’s rank correlation test. Figure 5 displays the Spearman correlation coefficients between each meteorological factor and ILI incidence across different lag periods. Darker shades in the figure indicate stronger absolute correlation coefficients, signifying a more substantial association between the factor and influenza activity. The results reveal that most meteorological factors exhibited some degree of correlation with ILI case counts at various lags, with the strongest associations generally observed at Lag^0^ (no lag). Monthly average atmospheric pressure showed significant positive correlations at Lag^0^ and Lag^1^ (*r* = 0.34 and *r* = 0.30, respectively; *p* < 0.05). In contrast, monthly average temperature, relative humidity, and total precipitation all demonstrated significant negative correlations at Lag^0^ (*r* = −0.30, *r* = −0.31, and *r* = −0.36, respectively; *p* < 0.05). Wind speed exhibited significant negative correlations at Lag^2^ and Lag^3^ (*r* = −0.31 and *r* = −0.34, respectively; *p* < 0.05), suggesting that its inhibitory effect on transmission manifests with a distinct lag (delayed effect).

With the exception of monthly average sunshine duration, all other examined meteorological factors showed statistically significant correlations with ILI case counts at one or more lag times. Atmospheric pressure, precipitation, and wind speed displayed particularly pronounced correlations. The strongest individual correlation observed was between monthly mean wind speed at a 2-month lag (Lag^2^) and ILI case counts.

The strongest associations were generally observed at Lag0 (no lag) for most meteorological factors, including temperature, humidity, and precipitation. Therefore, subsequent GAM and SARIMA analyses focused on Lag0 to capture the most immediate effects on ILI incidence.

#### 3.2.2 Generalized additive model (GAM) analysis

Based on the Spearman correlation results identifying Lag^0^ (no lag) as the most appropriate lag period, this study employed Generalized Additive Models (GAMs) to further analyze the relationship between meteorological factors and the number of ILI outpatient visits under Lag^0^ conditions. Univariate Analysis Results (Table 1): Monthly mean relative humidity showed a statistically significant association (edf = 2.59, *F* = 4.21, *p* = 0.0128), suggesting a potential nonlinear relationship with ILI case counts. Total precipitation exhibited a trend towards association, though it did not reach statistical significance (edf = 2.43, *F* = 1.82, *p* = 0.1632). Wind speed, sunshine duration, temperature, and atmospheric pressure demonstrated low *F*-values and non-significant *p*-values (*p* > 0.05), indicating no statistically significant associations in the univariate models.

**Table 1:** Relationship between influenza-like illness case numbers and meteorological factors based on GAM (Lag0).

Meteorological factors	Univariate analysis			Multivariate analysis		
	df	*F*-statistic	*p-*value	df	*F*-statistic	*p-*value
Humidity (%)	2.59	4.21	0.0128	5.12	2.4	0.00478
Precipitation (mm)	2.43	1.82	0.1632			
Wind speed (m/s)	2.52	0.81	0.4737			
Sunshine duration (h)	2.52	0.62	0.5773			
Temperature (°C)	2.63	0.36	0.7554			
Air pressure (mmHg)	2.58	0.30	0.7984			

The *F*-statistic is the test statistic used in Analysis of Variance (ANOVA); the *p*-value indicates the significance level; df represents degrees of freedom. Variables with variance inflation factor (VIF) > 5 were excluded from the multivariate model to avoid multicollinearity.

Prior to constructing the multivariate Generalized Additive Model (GAM), we assessed multicollinearity among meteorological variables by calculating variance inflation factors (VIFs), with VIF > 5 considered indicative of significant collinearity. Precipitation (VIF = 6.8), wind speed (VIF = 5.9), sunshine duration (VIF = 5.4), temperature (VIF = 8.2), and atmospheric pressure (VIF = 7.3) all exceeded this threshold, confirming substantial multicollinearity. Consequently, these variables were excluded from the final multivariate model to ensure stability and interpretability. After adjusting for the remaining factors, only monthly mean relative humidity (VIF = 2.1) remained a statistically significant predictor (edf = 5.12, *F* = 2.40, *p* = 0.00478), emerging as the sole meteorological variable independently associated with ILI outpatient visits. This finding indicates that, after controlling for other meteorological factors, relative humidity may exert a more independent and significant influence on the occurrence of ILI cases.

#### 3.2.3 SARIMA model analysis

**Figure 6:**
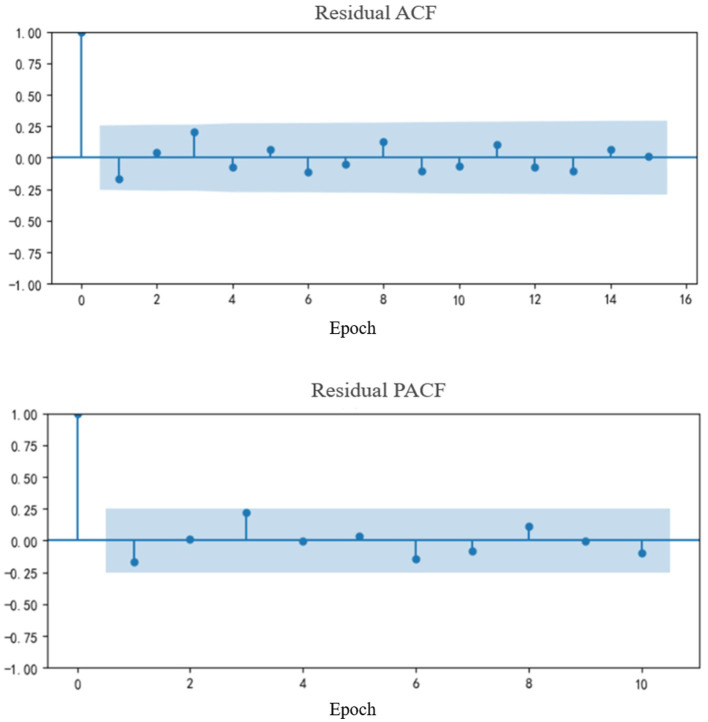
Residual plots of ACF and PACF.

The SARIMA model is a forecasting model used for time series analysis. It enables modeling and prediction of future trends based on historical data. This model comprehensively accounts for both seasonal and non-seasonal factors, making it particularly effective for capturing seasonal fluctuations and cyclical patterns within time series data. As established in previous analyses, the influenza epidemic in Tianjin exhibits distinct seasonal and cyclical characteristics. Consequently, the SARIMA model is well-suited for modeling and analyzing this data. Figure 6 illustrates the results after applying first-order seasonal differencing and first-order non-seasonal differencing to the original time series. The residual plots of both the Autocorrelation Function (ACF) and the Partial Autocorrelation Function (PACF) show no significant signs of abrupt cutoff (cutting off) or gradual decay (tailing off). This indicates that the differencing process effectively stabilized the volatility of the series.

**Figure 7:**
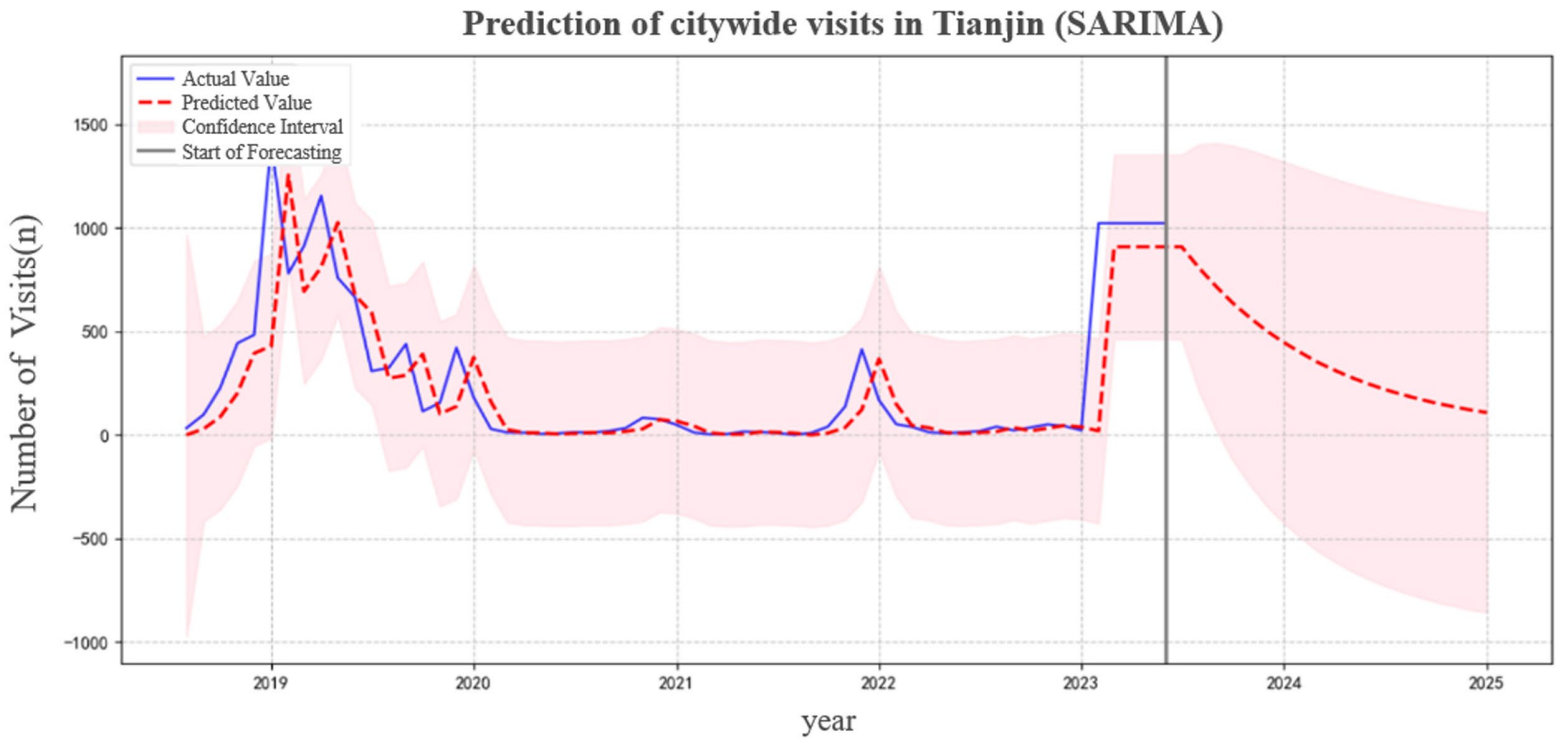
SARIMA modeling of the number of influenza-like illness cases among children in Tianjin from 2018 to 2023.

Leveraging the auto_SARIMA function in Python 3.8, the model parameters were initially identified automatically. This automated selection was further refined and optimized through manual adjustment based on examination of the Autocorrelation Function (ACF) and Partial Autocorrelation Function (PACF) residual plots. Following this process, the SARIMA(1,0,0)(0,0,0)_12_ model was ultimately selected as the optimal model for this study. Model performance evaluation showed that the model achieved a goodness-of-fit (*R*^2^) of 0.632 and a root mean square error (RMSE) of 27.33. These metrics indicate that the model achieved satisfactory goodness-of-fit and predictive performance. The predicted results generated by the model are presented in Figure 7.

## 4 Discussion

Through systematic analysis of Tianjin’s 2018–2023 pediatric ILI case and meteorological data, this study reveals spatiotemporal characteristics of pediatric influenza epidemics and their intrinsic associations with meteorological parameters. Results indicate that influenza epidemics in Tianjin exhibit significant winter clustering, with occasional peaks in spring, and notable regional heterogeneity: the central urban Hexi district has the highest incidence rates, while the suburban Ninghe district has the lowest. The reasons for this regional heterogeneity may involve two factors: first, the higher ambient humidity (4.6% higher than the average in Hexi District) and higher wind speed (1.2 m/s higher than the average in Hexi District) in Ninghe District may have a suppressive effect on viral transmission; and second, as the location of the Machang campus of the Tianjin Children’s Hospital, the higher availability of healthcare resources in Hexi District may have led to a significant increase in the child population density of this region. It is worth noting that children under 6 years of age accounted for more than 70% of the total number of cases, and this high prevalence requires multiple interpretations from a pediatric clinical perspective: the immature immune system, the anatomical characteristics of the respiratory tract (narrow airways, poor ciliomotor function), the group living environment (high-density congregation in childcare institutions), and the poor adherence to hand hygiene in the younger age group form the basis of susceptibility. It should be noted, however, that these district-level case proportions reflect healthcare-presenting cases at a single referral center rather than true community incidence rates; differential healthcare-seeking behavior and proximity to the hospital may contribute to the observed urban–suburban gradient independently of genuine epidemiological differences.

The SARIMA model offers distinct advantages in forecasting infectious disease dynamics by explicitly capturing seasonal periodicity—a critical feature for climate-driven pathogens like influenza. By incorporating multidimensional meteorological covariates through seasonal differencing and autoregressive components, SARIMA enhances predictive accuracy for epidemic resurgences while maintaining computational efficiency. Our meteorological-augmented SARIMA(1,0,0)(0,0,0)₁₂ model achieved robust predictive performance (*R*^2^ = 0.632) and transparent interpretation of environmental drivers. Compared with recent machine learning approaches—such as transformer-based models, attention-augmented LSTM networks, and hybrid deep learning architectures—which often require high-frequency data and suffer from limited interpretability, our SARIMA framework is particularly well-suited for pediatric surveillance settings with monthly data resolution (17, 18). Extensions of the SARIMA model incorporating exogenous covariates (SARIMAX) have shown improved fit in recent studies, aligning with our two-stage strategy of using GAM to identify dominant predictors before model specification (19, 20). Furthermore, the humidity-based early-warning trigger proposed in this study (relative humidity < 50% for ≥3 consecutive days) is consistent with emerging nowcasting frameworks and offers an operationally deployable tool for resource-limited health systems. Together, these findings underscore the complementary role of interpretable, meteorologically informed SARIMA models alongside more data-intensive approaches in building a layered influenza surveillance infrastructure.

In meteorological correlation analysis, GAM combined with Spearman testing reveal significant negative correlation between ILI incidence rates and relative humidity (strongest correlation at lag period = 0). This finding highly aligns with climatic characteristics during winter influenza peak periods—Tianjin’s dry winter climate. The dominance of Lag0 associations for relative humidity and temperature is consistent with published finer-resolution evidence [e.g., weekly lag analyses by Suntronwong et al. (21) and Marr et al. (22)], lending biological plausibility to our monthly findings and suggesting that the principal meteorological signal operates on a within-month timescale. Lei et al. (7) found that in temperate and subtropical regions, seasonal variations in indoor relative humidity significantly regulate influenza transmission. Suntronwong et al. (21) demonstrated significant correlations between relative humidity and influenza activity in Bangkok. Low humidity may promote virus transmission through multiple mechanisms: In low humidity environments, virus-containing droplets dehydrate to form aerosol nuclei, prolonging viral air retention time and transmission distance, and influenza viruses are active when the relative humidity is below 50%, especially between 20 and 35% (22); dryness of respiratory mucous membranes leads to impaired function of the mucus-ciliary clearance system, and weakening of the local intrinsic immune barrier (23, 24); and experimental studies have shown that the inactivating effect of antiviral proteins on influenza viruses is strongest under moderate humidity conditions, whereas extreme dryness significantly reduces the activity of such proteins (25). Notably, humidity effects on influenza transmission may show climate-dependent bimodal distribution. This study’s temperate region presents winter dry-high incidence patterns, while tropical region rainy season high humidity environments similarly correlate with outbreak occurrences (24, 26), suggesting need for establishing region-specific early warning models.

Influenza usually peaks in winter in temperate regions (27), and Chong et al. (6) showed that lower temperatures contribute to the spread of influenza A and B in temperate and subtropical regions, which is consistent with the conclusions reached in this study in 2018–2022. Temperature effects on pediatric influenza transmission mechanisms require interpretation from both viral biological characteristics and host behavioral pattern perspectives: (1) Viral level: low temperatures enhance the stability of the influenza virus envelope. Animal studies have shown higher influenza virus titers in guinea pigs exposed to low temperatures, with viruses exhibiting greater stability and longer half-lives. Transmission is most efficient at 5 °C and least efficient at 30 °C, indicating that influenza viruses have stronger transmission capabilities under low-temperature conditions (28, 29); (2) Host level: cold exposure can suppress nasopharyngeal interferon responses and reduce natural killer cell activity (30), while cold stimulation causes nasal mucosal vasoconstriction and increased mucus secretion, which together delay pathogen clearance (31); (3) Behavioral patterns: low temperatures encourage increased clustering of children in enclosed, heated environments and reduce ventilation in childcare centers and schools, significantly raising the likelihood of close contact (32, 33). In subtropical and tropical regions, seasonal characteristics become more complex and difficult to predict, with peak periods potentially occurring multiple times annually (34). Due to relatively small annual temperature variations in tropics, influenza virus epidemic patterns no longer simply follow unidirectional temperature influences, making viral epidemic peaks no longer limited to specific temperature ranges but presenting diversified temporal distributions (35). This study’s discovery of anomalous May 2023 peaks (average temperature 21.1 °C) suggests that under global warming contexts, viral evolutionary pressures (such as antigenic drift), population immunity level fluctuations, and post-pandemic social behavioral pattern changes may reshape traditional epidemic patterns, presenting new challenges for pediatric early warning system construction. It should also be noted that the 2023 surge partly reflects the well-documented post-pandemic rebound of influenza following 2 years of near-complete suppression under COVID-19 NPIs, and potentially improved influenza-specific attribution as SARS-CoV-2 testing became routine and ILI coding became more pathogen-selective. This temporal variation in the pathogen composition of ILI cases underscores the importance of interpreting ILI-based meteorological associations with caution, and highlights the value of integrating laboratory surveillance data in future studies.

For other meteorological parameters, Spearman correlation analysis results indicate monthly cumulative rainfall negatively correlates with ILI incidence rates. This finding is supported by recent studies in subtropical China, which have demonstrated that higher precipitation levels (>21 mm) exhibit a protective effect against influenza transmission (RR = 0.85), with the association persisting at a lag of 2 weeks (36), while two studies from Hong Kong and Hangzhou indicate significant positive correlations between rainfall and influenza activity (6, 37). No unified conclusions exist regarding relationships between rainfall and influenza incidence rates. Monthly average wind speed negatively correlates with ILI case numbers when lagged 2 months, suggesting lower wind speeds may increase influenza transmission risk, consistent with existing research conclusions from other regions (8, 38). Under Tianjin climatic conditions, lower wind speeds may extend viral droplet airborne residence time, thereby increasing infection probability when individuals are within droplet transmission range. Under higher wind speed conditions, although viral droplets disperse more quickly and travel greater distances, their airborne concentration and droplet particle size correspondingly decrease, potentially weakening infectivity (39). Limited literature exists regarding atmospheric pressure effects on influenza. This study finds significant positive correlation between ILI incidence rates and monthly average atmospheric pressure with lag effects, consistent with Guo et al.’s (40) finding of increased influenza cases under high pressure (above 1,005 Pa). This phenomenon may result from accelerated settling speeds of water droplets and dust in air under high atmospheric pressure conditions, thereby promoting viral transmission. Additionally, high atmospheric pressure is often associated with relatively dry air, which can cause nasal capillary vasoconstriction and damage to the nasal mucosa. This weakens the respiratory tract’s defense mechanisms, facilitating viral invasion and ultimately increasing the risk of influenza transmission (37).

Influenza transmission is influenced not only by meteorological factors but also by human prevention and control strategies. During the COVID-19 pandemic, non-pharmaceutical interventions (NPIs)—including school closures, mask-wearing, and social distancing—profoundly disrupted influenza dynamics, leading to the near disappearance of the disease in 2020–2021, followed by a sharp resurgence in spring 2023. This pattern aligns with the “immunity debt” hypothesis, which posits that reduced viral exposure during the pandemic increased population susceptibility, particularly among children. These shifts complicate the interpretation of long-term epidemiological trends and underscore the need for future models to incorporate structural breaks or NPI covariates to better inform pediatric public health strategies.

At prevention levels, influenza vaccination represents one of the most effective and cost-efficient strategies for influenza prevention (41). Over more than 60 years of global immunization practice and extensive research, influenza vaccines have consistently demonstrated good safety and efficacy. This is supported by recent systematic reviews and clinical trials confirming that both adjuvanted and non-adjuvanted influenza vaccines provide robust protection, with seroprotection rates exceeding 95% in young children. By reducing the risk of infection and related complications, vaccination also contributes to lowering community transmission (42). WHO recommends annual seasonal influenza vaccination for children aged 6–59 months. However, China’s total population vaccination rate remains only approximately 2%. Promoting influenza vaccines and improving vaccination rates remains a fundamental task for influenza prevention (43)_._ Therefore, promoting influenza vaccines and improving vaccination rates remains a fundamental task for influenza prevention in China. This study provides climatological evidence for optimized influenza vaccine immunization strategies: (1) The November humidity plunge period in Tianjin is highly compatible with the time of peak vaccine antibody (2–4 weeks after vaccination), and it is recommended that the window for centralized vaccination of children be shifted forward to mid-October. For the six central urban districts (such as Hexi), we recommend that the pediatric vaccination window be scheduled from October 1 to 15, corresponding to the period when monthly mean relative humidity typically falls below 55%, a threshold identified in our GAM analysis as associated with a significant rise in ILI cases. In contrast, the suburban districts (such as Ninghe) experience higher humidity and wind speed, which delay epidemic onset; thus, the recommended vaccination window may be extended to October 15 to 31. Furthermore, a humidity-triggered early warning system may be implemented, whereby a weekly average relative humidity drop below 50% can activate public health alerts and booster dose reminders, especially in high-risk regions; (2) In response to the contradiction between the vaccine protection cycle and viral variation, it is urgently needed to promote the research and development of broad-spectrum vaccines; (3) The current situation of the insufficient influenza vaccination rate in Chinese children calls for the establishment of a “doctor-school-society” joint vaccination system led by pediatricians.

The following limitations exist in this study: (1) This study used data from a single tertiary children’s hospital, which may introduce referral bias and may not fully reflect community-wide influenza transmission patterns. Although Tianjin Children’s Hospital serves as the primary pediatric referral center covering all districts, healthcare-seeking behavior and severity thresholds could influence case presentation. Future multicenter studies incorporating primary care and community surveillance are needed to validate and generalize our findings. (2) The use of ILI rather than laboratory-confirmed influenza may introduce outcome misclassification, particularly during periods when other respiratory viruses (e.g., RSV, SARS-CoV-2) co-circulate. Future studies should incorporate pathogen-specific data to improve diagnostic accuracy and better understand the interplay between viral etiology and meteorological drivers. (3) The 2020–2022 COVID-19 pandemic period introduced a structural break in the ILI time series driven by NPIs, which may confound the meteorological associations and inflate model goodness-of-fit; formal structural break analyses incorporating NPI intensity data are recommended for future work. (4) This study did not include comparative modeling with alternative approaches (e.g., ARIMAX, LSTM, Prophet), which would help contextualize the relative performance of our SARIMA model. Such comparisons are warranted in future work to guide model selection for public health practice. Additional limitations include failure to quantify the long-term effects of the COVID-19 pandemic on children’s immune imprinting, microenvironmental differences between outdoor meteorological data and children’s main activity scenarios (indoors/school buses, etc.), and the absence of interactive effects of air pollutants (such as PM2.5) with meteorological factors. Future multicenter pediatric cohort studies are needed to integrate individual exposure monitoring and immunohistological data to construct child-specific influenza prediction models.

## 5 Conclusion

This study characterizes pediatric ILI epidemiology in Tianjin, showing distinct winter seasonality, urban clustering, and disproportionate burden among children under 6 years. Key findings establish immediate relative humidity reduction (lag0) as the most sensitive early-warning biomarker for influenza surges and confirm elevated transmission risk under low-temperature conditions. The SARIMA(1,0,0)(0,0,0)_12_ model achieves superior predictive accuracy for ILI trends, enabling: enhanced early-warning systems, evidence-based resource allocation during epidemic peaks, and climate-adaptive pediatric vaccination strategies. These insights provide actionable frameworks for public health decision-making amid accelerating climate volatility.

## Data Availability

The raw data supporting the conclusions of this article will be made available by the authors, without undue reservation.
